# Syndapin is dispensable for synaptic vesicle endocytosis at the *Drosophila* larval neuromuscular junction

**DOI:** 10.1016/j.mcn.2008.10.011

**Published:** 2009-02

**Authors:** Vimlesh Kumar, Suneel Reddy Alla, K.S. Krishnan, Mani Ramaswami

**Affiliations:** a Smurfit Institute of Genetics and Trinity College Institute of Neuroscience, Lloyd Building, University of Dublin, Trinity College, Dublin 2, Ireland; b National Centre for Biological Sciences, Bangalore 560085, India; c Department of Molecular and Cellular Biology, Box 210106, University of Arizona, Tucson, AZ 85721, USA

## Abstract

Syndapin is a conserved dynamin-binding protein, with predicted function in synaptic-vesicle endocytosis. Here, we combine genetic mutational analysis with *in vivo* cell biological assays to ask whether *Drosophila* syndapin (Synd) is an essential component of synaptic-vesicle recycling. The only isoform of *Drosophila* syndapin (*synd*) is broadly expressed and at high levels in the nervous system. *synd* mutants are late-larval lethals, but fertile adult “escapers” frequently emerge. Contrary to expectation, we report that the Synd protein is predominantly postsynaptic, undetectable at presynaptic varicosities at *Drosophila* third-instar larval neuromuscular junctions. Electrophysiological and synaptopHluorin imaging in control, synd-deficient or synd-overexpressing motor neurons reveals that synd is dispensable for synaptic-vesicle endocytosis. Our work in *Drosophila* leads to the suggestion that syndapin may not be a general or essential component in dynamin-dependent synaptic-vesicle endocytosis.

## Introduction

Endocytosis involves membrane bending and its scission to form vesicles. This involves concerted action of various classes of protein including the large GTPase, dynamin. Several dynamin interacting, BAR/F-BAR domain containing proteins like endophilin, amphiphysin and syndapins have been implicated in the process of vesicle endocytosis. (Takei et al., 1999; Farsad et al., 2001; Anggono et al., 2006). The N-terminal module of these proteins can bind and deform the lipid membrane whereas the C-terminal SH3 domain can mediate interactions with other proteins. The membrane-tubulating activity of several BAR domain containing proteins, including the F-BAR domain protein syndapin, has been demonstrated both *in vitro* and in cell cultures (Itoh et al., 2005).

Syndapins were first identified as the binding partners for fly dynamin and/or as phosphoproteins upregulated during neuronal development (Plomann et al., 1998; Qualmann et al., 1999). Syndapin's SH3 domain binds proline-rich domains (PRD) of dynamin as well as the actin-regulatory protein WASp (Kessels and Qualmann, 2002). These initial observations, together with other lines of additional data (Merrifield et al., 1999; Merrifield et al., 2002; Merrifield et al., 2004) led to a model in which syndapin facilitates membrane internalization in dynamin-mediated synaptic vesicle recycling. Two recent studies provide experimental support for this model. Anggono et al. showed that the dephosphorylated form of dynamin, which occurs transiently following synaptic vesicle exocytosis, is not only required for compensatory endocytosis in synaptosomes, but also for syndapin binding (Anggono et al., 2006). Andersson et al. showed that an axonally loaded antibody against syndapin not only accumulates at lamprey nerve terminals, but also specifically blocks synaptic-vesicle recycling (Andersson et al., 2008).

After confirming the conservation of biochemical activities of *Drosophila* syndapin (Synd) as well as its neural expression, we asked: a) whether Synd is a presynaptic protein; and b) if it is required for synaptic-vesicle endocytosis at the *Drosophila* larval motor synapses. Contrary to prevailing models and our initial expectation, we find that Synd is largely postsynaptic and is not required for efficient synaptic-vesicle endocytosis at the motor terminals.

## Results

### Molecular organization of syndapin locus

**Fig. S1:**
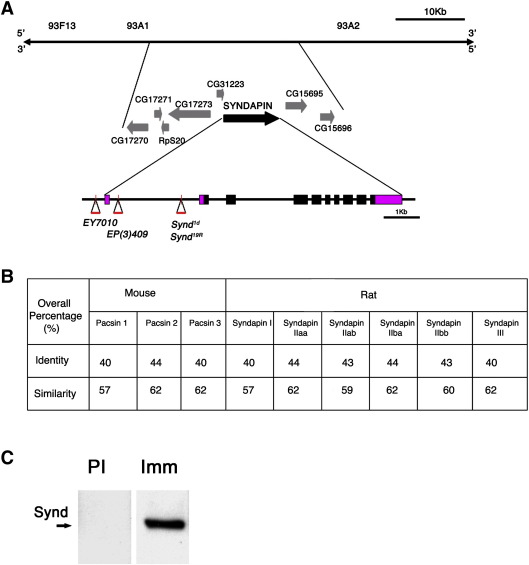
Molecular organization of the Drosophila syndapin locus in wild-type and transposon induced alleles, and the embryonic expression pattern for Synd. (A) Synd gene is located at 93A1 of cytological position at third chromosome and consists of ten exons. The 5´ and 3´ untranslated regions are shown in pink. The open reading frame is depicted in black. The position of various P-elements is marked. The translational start site is located in the second exon. (B) Percentage identity and similarity between *Drosophila* syndapin and its mammalian orthologs. (C) Western blot showing that syndapin antiserum (Imm), but not pre-immune serum (PI) recognizes a 57 kDa protein in fly head extracts.

**Fig. 1:**
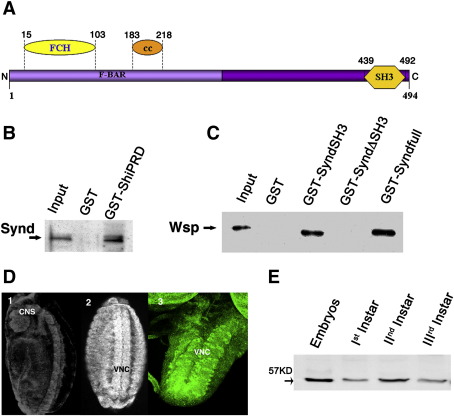
Conserved structure and biochemical interactions of *Drosophila* syndapin. (A) Like its mammalian orthologs, *Drosophila* syndapin has an N-terminal F-BAR domain comprising FCH and coiled-coil (CC) regions and a C-terminal SH3 domain. (B) Synd interacts with proline-rich domain of shibire. Bacterially expressed GST-shibire PRD pulls down syndapin from *Drosophila* head lysates, as seen in western blot analysis of pulled down proteins probed with anti-Synd antibody. (C) Various domains of syndapin were expressed as GST-tagged fusion protein and binding proteins in fly head lysate pulled down using Glutathione sepharose beads. Western blot of pulled down proteins in each case were probed with anti-Wsp antibody. (D) Embryos (D1 and D2) and third instar larval ventral nerve cord (D3) of *Drosophila* stained with anti-Synd antibody. Note that Synd although enriched in central nervous system and ventral nerve cord, it is expressed ubiquitously in embryos. (E) Western blots of fly lysate from different stages of *Drosophila* development. A single band of about 57 kDa was observed at all stages analyzed.

*Drosophila* syndapin (*synd*) was identified by analysis of the *Drosophila* genome sequence for homologs of mammalian genes implicated directly or indirectly in exocytosis or endocytosis of synaptic vesicles (Lloyd et al., 2000). Unlike mammals, which have three genes for syndapin, the *Drosophila* genome contains a single gene for syndapin (Supplementary Fig. S1A). The *synd* locus produces a single transcript of about 3.2 kb comprising 10 exons. The *Drosophila* syndapin shares an overall sequence similarity of about 55% to its mammalian orthologs (Supplementary Fig. S1B). Conceptual translation of this experimentally confirmed *synd* ORF predicts a protein of 494 amino acids with domain organization remarkably similar to vertebrate syndapins (Fig. 1A): thus, it consists of the N-terminal F-BAR domain with about 49% identity and a C-terminal SH3 domain with over 65% identity to mammalian syndapin 1.

### Conserved biochemical properties of *Drosophila* syndapin

Previous studies have shown that mammalian syndapins interact *in vitro* with the PRD-domain proteins, dynamin and N-WASp, which have been associated with function in endocytosis (Qualmann et al., 1999; Qualmann and Kelly, 2000; Anggono et al., 2006). We used GST-pulldown to confirm that these properties were conserved for *Drosophila* syndapin. Using a highly specific anti Synd antibody raised against a bacterially expressed Synd (N-terminal, 1–377 amino acids) fusion protein (Supplementary Fig. S1C), we asked whether the PRD-domain of *Drosophila* dynamin (Shi-PRD) immobilized on glutathione-sepharose beads would pull down Synd from *Drosophila* adult head lysates. As shown in Fig. 1B, the Shi-PRD column specifically retained syndapin. We similarly asked whether *Drosophila* WASp (Wsp) would also interact with Synd. Column-bound Synd could pull down Wsp from fly head extracts through a mechanism that required the SH3 domain of syndapin (Fig. 1C). Significantly, neither the N-terminal F-BAR domain of syndapin nor control, immobilized GST pulled down the Wsp protein. Taken together, these data indicate that at least these two well-described biochemical properties of mammalian syndapins are conserved in Synd.

### Syndapin is localized postsynaptically at the third instar larval neuromuscular junction

Synd is not a neural specific protein. In *Drosophila* embryos, the protein was more or less ubiquitously present in early stages of embryonic development. At Stage 16, the protein is enriched in the nervous system of the embryos (Fig. 1D). In third-instar larvae, the syndapin immunoreactivity was concentrated in, though not restricted to the ventral nerve cord (Fig. 1D). Western-blot analyses indicate single 57 kDa form of Synd in adult head as well as all *Drosophila* developmental stages analyzed (Fig. 1E and Supplementary Fig. S1C).

**Fig. S2:**
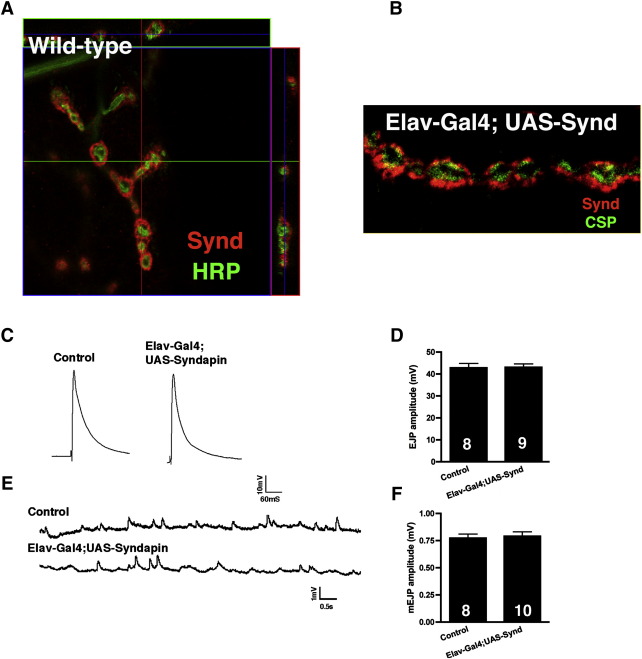
Synd overexpression in motor neurons does not result in visible presynaptic Synd and synaptic transmission is normal in synd overexpressors. (A) Representative orthogonal planes; XZ (red line), XY (green line) and YZ (blue line) from a bouton double-labeled for synadpin (red) and HRP (green), confirming that syndapin is postsynaptic and enriched in SSRs. (B) Single confocal section of NMJ overexpressing syndapin in motor neurons, double labeled with syndapin (red) and CSP (green). Note that synd is not trafficked to boutons. (C–F) Representative traces of (C) evoked synaptic potentials from control and Synd overexpressing animals; (D) average EJP amplitudes for the indicated genotypes; (E) representative traces of mEJP from control and syndapin overexpressing synapses and (F) histogram showing average mEJP amplitudes for the indicated genotypes. mEJP amplitude was 0.77 ± 0.04 mV in controls compared to 0.79 ± 0.043 mV in *Elav-Gal4; UAS-Synd*, P > 0.75; and EJP amplitude was 42.8 ± 2.0 mV in controls compared to 43.1 ± 1.54 mV in *Elav-Gal4; UAS-Synd*, P > 0.9. Number in histogram indicate number of animals analyzed. Error bar represents standard error of the mean (s.e.m.).

**Fig. 2:**
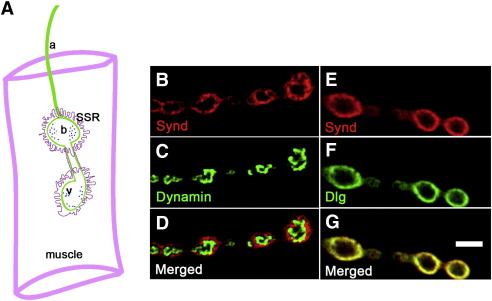
At the *Drosophila* larval NMJ, syndapin is a postsynaptic protein highly enriched in the subsynaptic reticulum. (A) Cartoon of the *Drosophila* larval NMJ showing the presynaptic axon terminal (green) embedded in a postsynaptic, tubulolamellar membrane system (pink) called the subsynaptic reticulum (SSR). (B–D) Synd does not colocalize with presynaptic dynamin. Single confocal section showing localization of Synd (B, red), dynamin (C, green) and the difference in localization of both proteins (D) within type I boutons. (E–G) Synd colocalizes with Dlg, a marker of postsynaptic SSR. Single confocal section of type I boutons of a wild-type larva, double labeled with anti-Synd (red) and anti-Dlg (green) antibodies. (G) Merged image of (E) and (F). Scale bar represents 5 μm for B–G.

Current models for syndapin function in neurons are significantly based not only on its binding to dynamin, but also on its presumed presynaptic localization (Qualmann and Kelly, 2000). To directly determine syndapin's subcellular distribution, we analyzed Synd localization at the *Drosophila* larval neuromuscular junctions (NMJ). Analyses of double-labeled NMJ synapses showed no colocalization with presynaptic dynamin (Figs. 2B–D) which marks sites of synaptic-vesicle endocytosis (endocytic hot-spots) at the *Drosophila* NMJ (Estes et al., 1996; Roos and Kelly, 1998). The absence of any obvious colocalization between dynamin and Synd suggested that Synd is either absent, or present at very low levels in the presynaptic compartment. This suggestion is supported by analysis of Synd localization relative to HRP protein, a presynaptic membrane marker, which shows Synd to be present around, rather than within, presynaptic boutons (Supplementary Fig. S2A). Since, we could not rule out the possibility of small amount of Synd not being detected by immunofluorescence in presynaptic terminals, we overexpressed a syndapin transgene in motor neurons. However, expressing this transgene did not cause any enrichment of Synd protein at motor terminals (Supplementary Fig. S2B). This suggests that synd is not normally trafficked to boutons.

In contrast, Synd co-localized substantially with Dlg, a protein previously localized to the subsynaptic reticulum (SSR) (Lahey et al., 1994) (Figs. 2E–G). Thus, Synd is a predominantly postsynaptic protein. This conclusion is consistent with a recent study showing that Syndapin1/Pacsin1 localizes to the postsynaptic dendritic spines in rat hippocampal neurons in culture (Perez-Otano et al., 2006).

Together these localization studies suggest that Synd may not have important presynaptic functions. To resolve this, we created animals with reduced or increased levels of Synd and asked how these perturbations affected synapse function.

### Isolation and characterization of *synd* mutants

**Fig. 3:**
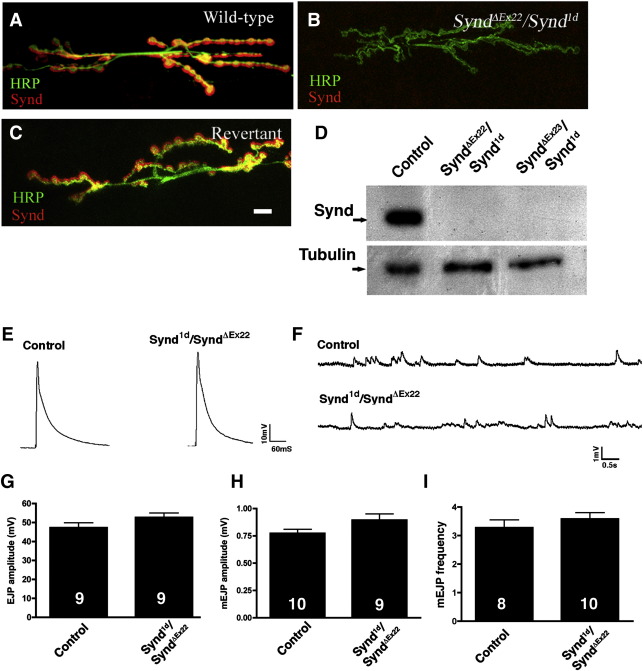
Syndapin loss-of-function mutants show normal synaptic transmission. (A–C) NMJ synapses of third-instar larvae co-stained for axonal plasma membrane (anti-HRP, green) and syndapin (red) of: (A) wild type, (B) *synd* heteroallelic combination (*synd^ΔEx22^/synd^1d^*) and (C) syndapin revertant animal (obtained from the precise molecular excision of P-element of *synd*^*1d*^). A strong syndapin immunoreactivity around boutons of wild type animals is absent (reduced to background levels) in the *synd* mutants. Note that syndapin immunoreactivity at the NMJs reverts to wild-type levels in Synd revertants. (D) Western blot analysis of lysates from third instar larval fillets of control and *synd* mutant confirming that the syndapin protein is absent or dramatically reduced in the mutants. (E–I) Representative traces of (E) evoked synaptic potentials (F) spontaneous mEJP from control and *synd* (F2-generation) mutant animals; (G) average EJP amplitude (H) average mEJP amplitude and (I) average mEJP frequency of indicated genotypes. Numbers in histogram indicate number of animals analyzed. Error bar represents standard error of the mean (s.e.m.). Scale bar represents 10 μm for A–C.

We employed standard genetic techniques to generate loss-of-function mutations in the *synd* gene. Transposon-insertion lines, EP409 and EY7010 in Synd were identified and their genomic positions, respectively in the first intron and 100 bp upstream of the putative transcriptional start site, identified by sequencing the isolated flanking DNA. EP409 and EY7010 lines were viable as homozygotes, consistent with our observation that they allowed robust Synd expression (data not shown). To generate severe loss-of-function alleles in *synd*, we mobilized P-elements in EP409 or EY7010 and screened excision lines for the absence/reduction of Synd expression by immunostaining of larval NMJs. Two new P-alleles: *synd*^*1d*^ and *synd*^*19R*^ (from EP409 remobilization) and 2 deletion lines: *synd*^*ΔEx22*^ and *synd*^*ΔEx23*^ (from EY7010 remobilization) recovered showed absence of syndapin immunoreactivity at the NMJs and on western blots (Figs. 3A–D). The *synd* P-insertion were pupal lethal with few adult escapers. The escapers were behaviorally normal, often producing mutant progeny that died at the larval/pupal stage — suggesting that maternally provided Synd protein was not required for viability in early embryonic/larval stages.

The position of P-element in *synd*^*1d*^ was ascertained by inverse PCR. Remobilization of the P-element in *synd*^*1d*^ allowed viable revertants (from precise molecular excisions) to be obtained that now showed normal syndapin immunostaining at the NMJs, further confirming that the lethality in the mutant strains is caused by disruption of the *synd* locus (Fig. 3C). The absence of detectable protein by immunostaining and western-blot analyses of third-instar animals suggested syndapin alleles *synd*^*1d*^ and *synd*^*ΔEx22*^ to be very strong hypomorphs, or possibly protein nulls.

### Synaptic transmission is normal in syndapin loss-of-function allele and overexpressors

Our observation that Synd is predominantly postsynaptic did not exclude the possibility that a minor amount of Synd is present presynaptically and is sufficient for function in synaptic vesicle cycling. To directly address this possibility, we analyzed synaptic transmission and rates of synaptic-vesicle exocytosis and endocytosis in animals with genetically altered *synd* function. Bouton number and axon branching patterns were normal in *synd* mutants (*synd^1d^/synd^ΔEx22^*); in addition, the organization of synaptic-vesicle clusters to the extent observed by immunohistochemical and confocal microscopy studies appeared normal in the mutants (data not shown). The average number of boutons and axon branches per synapse, and the average fluorescence of various glutamate receptors around each bouton were not significantly different from the control animals (data not shown).

**Fig. 4:**
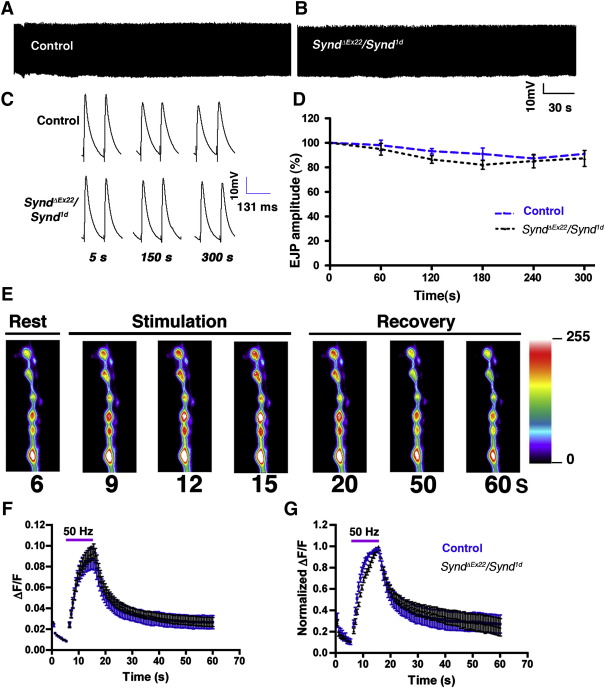
Syndapin loss-of-function mutants show normal rate of endocytosis (A, B) Synaptic depression was not observed in Synd mutants during high-frequency nerve stimulation. Continuous EJP recordings from (A) control and (B) *synd* heteroallelic larvae (*synd^ΔEx22^/synd^1d^*) stimulated at 10 Hz for 5 min. (C) Representative traces of EJPs at indicated time points during 10 Hz stimulation in 1.5 mM Ca^2+^ containing HL3 saline. (D) Normalized EJP amplitudes in control (black lines) and *synd* heteroallelic larvae (*synd^ΔEx22^/synd^1d^*) stimulated at 10 Hz for 5 min. No significant difference in EJP amplitude was observed at any time points. (E) Representative wild-type SpH responses to 50 Hz stimulation before, during and after stimulation. (F) Indistinguishable SpH responses in control (blue, *n* = 25 boutons, 5 animals; *Elav*^*3E*^, *UAS-SpH*, *synd^1d^/+*) and mutant synapses (black, *n* = 25 boutons, 6 animals; *Elav*^*3E*^, *UAS-SpH*, *synd^1d^/synd^ΔEx22^*) to a 50 Hz, 10 s stimulus train in HL3 containing 2.0 mM Ca^2+^. The horizontal bar represents the duration of stimulus. SpH fluorescence intensity was normalized to the image prior to the onset of stimulus. (G) Same data as in F, however, values normalized to the peak Δ*F*/*F* intensity, for visual comparison of endocytosis rate after the stimulus train. Error bars are standard error of the mean (s.e.m.).

Electrophysiological analyses showed “F2-generation” syndapin mutants (*synd^1d^/synd^ΔEx22^*) to be indistinguishable from controls in basic aspects of synaptic transmission (Figs. 3E–I). Thus, *synd^1d^/synd^ΔEx22^* have normal: a) mEJP amplitude (0.92 ± 0.05 mV compared to 0.80 ± 0.07 mV in controls; *P* > 0.19); b) EJP amplitude (49.8 ± 2.3 mV compared to 46.55 ± 2.4 mV in controls; *P* > 0.34) and mEJP frequency (3.6 ± 0.22 compared to 3.3 ± 0.3 in controls; *P* > 0.38). In addition, the observation that mutant synapses can sustain 5 min of high frequency (10 Hz) stimulation in 1.5 mM Ca^2+^ would not be expected if *synd* mutants have substantially reduced rates of synaptic-vesicle recycling (Figs. 4A–D) (Stimson et al., 2001; Verstreken et al., 2002; Marie et al., 2004). Synd overexpression in the motor neuron also has no significant effect on synaptic transmission (Supplementary Figs. S2C–F): thus, mEJP amplitude (0.77 ± 0.04 mV in controls compared to 0.79 ± 0.043 mV in *Elav-Gal4*; *UAS-Synd*, *P* > 0.75) and EJP amplitude (42.8 ± 2.0 mV in controls compared to 43.1 ± 1.54 mV in *Elav-Gal4*; *UAS-Synd*, *P* > 0.9) were indistinguishable between control and Synd overexpressing motor neurons. These observations indicate that syndapin is largely dispensable for synaptic transmission, at least under the conditioned tested in our experiments.

### Rate of synaptic vesicle endocytosis is normal in synd loss-of-function alleles

Since synaptic transmission can be normal even if the rate of vesicle internalization is compromised, we tested whether syndapin mutants are compromised for vesicle internalization using quantitative assays for rates of vesicle endocytosis under high-frequency nerve stimulation. We measured the kinetics of vesicle endocytosis in experimental and control animals using synaptopHluorin (SpH) imaging (Miesenbock et al., 1998; Poskanzer et al., 2006). We expressed SpH transgene pre-synaptically in syndapin mutants (*Elav^3E1^-Gal4*, *UAS-SpH*, *synd^1d^/synd^ΔEx22^*) (Witzmann et al., 2003) and control (*Elav^3E1^-Gal4*, *UAS-SpH*, *synd^1d^/+*) animals. The NMJ synapses were stimulated at 50 Hz for 10 s and the change in fluorescence normalized to base line fluorescence (Δ*F*/*F*) was calculated (Figs. 4E–G). The kinetics of vesicle recycling in *synd* mutants and controls were not significantly different (Fig. 4G). The rates of reformation of normal pH synaptic vesicles were *τ* = 7.9 ± 0.67 s and 8.3 ± 0.67 s for *Elav^3E1^-Gal4*, *UAS-SpH*, *synd^1d^/synd^ΔEx22^* and *Elav^3E1^-Gal4*, *UAS-SpH*, *synd^1d^/+*, respectively when fit to a single exponential function (*P* > 0.72).

Taken together, our immunocytochemical, electrophysiological and live SpH imaging data indicate that syndapin is dispensable for synaptic transmission and synaptic-vesicle endocytosis at the *Drosophila* motor terminals.

## Discussion

Surprising in the context of our initial guiding hypothesis, our data suggest that syndapin has little, if any role in synaptic-vesicle recycling at the *Drosophila* motor synapse. Several previous observations have contributed to a model in which syndapin facilitates endocytosis. First, and most significant, Synd binds dynamin *in vitro* (Qualmann et al., 1999; Kessels and Qualmann, 2002). Second, Synd appears to function in endocytosis. Overexpression of syndapin SH3 domain inhibits receptor-mediated endocytosis in HeLa cells arguing, albeit weakly, for a direct role in membrane internalization (Qualmann and Kelly, 2000; Kessels et al., 2006), a hypothesis supported by the observed colocalization of WASp, ARP2/3 and actin at the sites of clathrin-mediated endocytosis (Merrifield et al., 2002; Merrifield et al., 2004). Third, a role in endocytosis is also supported by recent studies showing that internalization of NR3A-containing N-methyl-d-aspartate receptors into postsynaptic endosomes requires interactions with, and function of Pacsin-1 (Perez-Otano et al., 2006).

Is syndapin a specialized or general regulator of endocytosis? The relatively well-supported premise that syndapin facilitates endocytosis has been extended to a model in which syndapin is postulated to be a critical requirement for synaptic-vesicle recycling in presynaptic terminals (Qualmann et al., 1999; Simpson et al., 1999). This proposal has been recently buttressed by a series of experiments documenting tight correlations between dynamin dephosphorylation, syndapin binding and synaptic-vesicle endocytosis (Anggono et al., 2006). In addition, a presynaptically delivered anti-syndapin antibody has been shown to inhibit synaptic-vesicle recycling in lamprey terminals (Andersson et al., 2008). However, experiments to assess presynaptic consequences of *precise* Synd perturbation *in vivo*, or convincingly localize Synd to presynaptic terminals, have not yet been performed. These lacunae are addressed by our present experiments.

Two critical observations presented here are inconsistent with a requirement for Synd in presynaptic function. First, Synd is predominantly a postsynaptic molecule at the *Drosophila* NMJ. Whereas proteins implicated in synaptic-vesicle recycling are enriched in the presynaptic varicosities at the *Drosophila* neuromuscular junctions (Littleton et al., 1993; Estes et al., 1996; Roos and Kelly, 1998; Qualmann et al., 1999; Rikhy et al., 2002; Coyle et al., 2004), Synd is undetectable within presynaptic boutons and, instead, highly enriched in the postsynaptic region. This postsynaptic localization in *Drosophila*, is highly consistent with recent studies that describe predominantly postsynaptic expression of mammalian syndapin1, as well as colocalization with postsynaptic density proteins in dendritic spines of rat hippocampal neurons (Perez-Otano et al., 2006). Thus, the *in vivo* localization of Synd does not easily support a primary function in synaptic-vesicle recycling.

Second, our functional analyses of Synd-deficient or Synd overexpressing nerve terminals argue against the possibility that low levels of presynaptic Synd poorly visualized by antibody labeling make substantial contribution to synaptic vesicle recycling. Electrophysiological studies show that loss of D-synd from synapses does not affect quantal size or quantal content of transmitter release evoked by either single stimuli or prolonged 10 Hz trains of nerve stimulation. Optical measurements show that rates of synaptic-vesicle exocytosis and reformation are indistinguishable at Synd-deficient and control nerve terminals.

We cannot exclude the possibility that presynaptic Synd may be present and regulate synaptic vesicle recycling in specific neuronal subtypes. However, our data argue against a general requirement for Synd in synaptic-vesicle recycling *in vivo*. Instead, it suggests that Synd may prove to be a cargo- or context-specific regulator of endocytosis (Tosoni et al., 2005). However, as some mammalian proteins — e.g. amphiphysin appear required for synaptic-vesicle recycling in mammals, but not in *Drosophila*, it is conceivable that Synd functions differently in insect and mammalian neurons (Razzaq et al., 2001; Zelhof et al., 2001; Di Paolo et al., 2002). More extensive and careful studies in different preparations are required to address the biological context in which syndapin function, especially its involvement in the synaptic vesicle endocytosis.

## Experimental methods

### Fly stocks and generation of mutants

Flies were maintained at 25 °C unless otherwise stated. All stocks and crosses were grown in standard corn meal medium. EP409 and EY7010 were obtained from Exelixis Inc., USA and Hugo Bellen, Baylor College of Medicine USA, respectively. *synd*^*1d*^ was obtained by remobilizing the transposon of EP409 by using Δ2–3 transposase. Genomic rescue of 3′-end with respect to EP-transposon using iPCR revealed that P-element in *synd*^*1d*^ and *synd*^*19R*^ disrupts the first intron of *synd*. Viable revertants of *synd*^*1d*^ were recovered by excising the P element in this line. *synd*^*ΔEx22*^ was a deletion line obtained by mobilizing P-element of EY7010. UAS-SpH flies were obtained from Dr. Graeme Davis, UCSF, USA. All other fly stocks are a part of the Tata Institute of Fundamental Research or Ramaswami laboratory stock collections.

### Antibodies and immunochemistry

Larval fillets were stained as described previously (Rikhy et al., 2002). Polyclonal anti-syndapin antibodies were raised in rat and rabbit against the N-terminal (SyndΔSH3, aa1–377) of the protein. Affinity purified anti-syndapin antibody was used at 1:50 dilution for immunostaining. Polyclonal rabbit anti-Dlg was a gift from Vivian Budnik and was used at 1:1000 dilution. Polyclonal anti-dynamin (Estes et al., 1996) was used at 1:200. Secondary antibodies coupled to Alexa Fluor 488 or Alexa Fluor 555 (Molecular Probes) were used at 1:400 dilution.

### GST-pull downs

Various GST-tagged domains of Synd or Shi-PRD were incubated with fly head lysates for 1 h at 4 °C. All pulldown experiments were performed in the presence of 20 mM Tris Cl; pH 7.5, 100 mM NaCl, 0.5% NP40, 0.5 mM EDTA, 0.5 mM PMSF and EDTA-free complete protease inhibitor tablets (Roche). Beads were washed extensively, bound proteins eluted in Laemilli buffer (2% SDS; 10% glycerol; 62.5 mM Tris–Cl, pH 6.8; 100 mM DTT, 0.1% bromophenol blue) and analyzed on western blots (also see Supplemental methods).

### Generation of UAS-syndapin transgenic flies

To generate the syndapin transgenes, PCR was performed using the primers SyndF: 5′-CAAGAATTCATGTCCCACCACAGCGATG-3′ and SyndR: 5′-ATTGCGGCCGCTTACGCGGTCTCCACATAG-3′ using cDNA as template. The PCR product containing Synd ORF was cloned at EcoRI and Not I site in pUAST. We also generated constructs of pUAST-syndapin by cloning the entire EST clone (LD46328) containing 5′ and 3′ UTRs at EcoRI site in pUAST. The embryonic transformation of *Drosophila* was performed by Genetic Services Inc, MA, USA. Several transgenes were obtained and all of them expressed Synd protein at high levels.

### Electrophysiology

Evoked junctional potentials (EJPs) were obtained from muscle 6 of A2 or A3 hemi-segment of wandering third-instar larvae, dissected in HL3 saline (70 mM NaCl, 5 mM KCl, 20 mM MgCl2, 10 mM NaHCO3, 5 mM Trehalose, 115 mM Sucrose and 5 mM HEPES; pH 7.3) containing 1.5 mM Ca^2+^. Briefly, electrodes were pulled on a Sutter P 2000 (Sutter Instrument Co.) from 1 mm O.D. TW100F-4 borosilicate glass (WPI). When filled with 3 M KCl the recording electrode had a resistance of 15–20 MΩ. A suction electrode, made from the same glass and filled with HL3 was used to stimulate the nerve innervating muscle segment A2 or A3. EJPs were recorded by stimulating at 4–6 mV at 1 Hz. Synaptic depression was achieved by stimulating at 10 Hz for 5 min.

Membrane potential and evoked EJPs were recorded using an AxoClamp 2B amplifier (Axon instruments) in bridge mode. Recordings were only collected from preparations where the membrane potential was between − 60 mV and − 66 mV. The stimulus train was generated with a S88 Grass stimulator and an SIU5 stimulus isolator. Signals were digitized via a 16 bit Digidata 1322A A to D converter (Axon instruments) at a sampling rate of 50 kHz and low pass filtered at 1 kHz. Data were processed and analyzed with pClamp (Axon Instruments). Miniature EJPs (mEJP) observed over a 30 s time period were analyzed using Mini Analysis software (Synaptosoft Inc., Decatur, GA). Recordings from at least 7 animals of each genotype were used for data analysis. Data were analyzed using unpaired *t*-test.

### SynaptopHluorin Imaging

Imaging of synaptopHluorin were performed as described previously (Poskanzer et al., 2006). Briefly, images were captured using a Zeiss Axioscope 2 microscope with a water immersion lens (W Plan Apo 63× 1.0VIS/IR). The preparation was illuminated by a 175 W Xenon arc lamp in a DG4 wavelength switcher containing a GFP filter set (Sutter Instrument Co). A series of images were acquired at 500 ms intervals using a Cascade 512b cooled CCD camera (Photometrics). The DG4 and the camera were controlled by of Metafluor Ver 6.1 software (Molecular Devices Corporation, Downingtown, PA USA). Normalized changes in average SpH fluorescence (Δ*F*/*F*) at each time point were quantified by defining a region of interest (ROI) that consisted of at least 25 type 1 boutons and subtracting the intensity of the ROI of an image immediately before stimulus from the image at each subsequent time point. The *τ* value for SpH decay kinetics was calculated by fitting the fluorescence decay following stimulation to a single exponential function using Graphpad PRISM^®^ 4. Only boutons in muscle 6/7 of A2 or A3 segment were used for analysis.
