# Supplementary material for: Syndapin is dispensable for synaptic vesicle endocytosis at the Drosophila larval neuromuscular junction
Source: Mol Cell Neurosci. 2009 Feb;40(2-3):234–41. doi: 10.1016/j.mcn.2008.10.011 (PMC2697329; doi:10.1016/j.mcn.2008.10.011)
Supplement: Supplemental methods [file mmc1.doc]

**SUPPLEMENTAL FIGURE LEGENDS**

**Figure S1: Molecular organization of the *Drosophila* syndapin locus in wild-type and transposon inuced alleles, and the embryonic expression pattern for Synd.**

(**A**) Synd gene is located at 93A1 of cytological position at third chromosome and consists of ten exons. The 5′ and 3′ untranslated regions are shown in pink. The open reading frame is depicted in black. The position of various P-elements is marked. The translational start site is located in the second exon.

(**B**) Percentage identity and similarity between *Drosophila* syndapin and its mammalian orthologs.

(**C**) Western blot showing that syndapin antiserum (Imm), but not pre-immune serum (PI) recognizes a 57 KDa protein in fly head extracts.

**Figure S2: Synd overexpression in motor neurons does not result in visible presynaptic Synd and synaptic transmission is normal in synd overexpressors.**

(**A**) Representative orthogonal planes; XZ (red line), XY (green line) and YZ (blue line) from a bouton double-labeled for synadpin (red) and HRP (green), confirming that syndapin is postsynaptic and enriched in SSRs.

(**B**) Single confocal section of NMJ overexpressing syndapin in motor neurons, double labeled with syndapin (red) and CSP (gren). Note that synd is not trafficked to boutons.

(**C-F**) Representative traces of (**C**) evoked synaptic potentials from control and Synd overexpressing animals; (**D**) average EJP amplitudes for the indicated genotypes; (**E**) representative traces of mEJP from control and syndapin overexpressing synapses and (**F)** Histogram showing average mEJP amplitudes for the indicated genotypes. mEJP amplitude was 0.77 + 0.04 mV in controls compared to 0.79 + 0.043 mV in *Elav-Gal4*; *UAS-Synd*, P> 0.75; and EJP amplitude was 42.8 + 2.0 mV in controls compared to 43.1 + 1.54 mV in *Elav-Gal4*; *UAS-Synd*, P> 0.9.

Number in histogram indicate number of animals analyzed. Error bar represents standard error of the mean (s.e.m).

**SUPPLEMENTAL METHODS**

**GST-Pulldown experiment**

Syndapin constructs representing the full length protein (Syndfull), the Syndapin N-terminal excluding the SH3 domain (SyndΔSH3) and the Syndapin SH3 domain excluding the FCH domain (SyndSH3) were amplified by PCR from cDNA made from fly head RNA. The PCR primers used were SyndfullExpF (5′-CAAGGATCCATGTCCCACCACAGCGAT-3′), SyndΔSH3ExpR (5′-AAGCTTGGCTTCCGATGTGGTGAC-3′), SyndSH3ExpF (5′-CGGATCCATGGCCATGAATTGGC-3′), SyndSH3ExpR (5′-AAGCTTCGCTTACGCGGTC

TCCAC-3′). Primer pairs: SyndfullExpF/SyndSH3ExpR, SyndfullExpF/SyndΔSH3ExpR and SyndSH3ExpF/SyndSH3ExpR were used to amplify full-length Syndapin, SyndapinΔSH3 and SyndapinSH3 domains respectively. The amplicons were restriction digested with BamHI and HindIII and ligated into the pGEX-KG plasmid at the same site to obtain GST-tagged recombinant proteins. All GST-tagged proteins were expressed in *E. coli* and purified using glutathione (GSH) -agarose beads (Amersham Biosciences) according to the manufacturer’s instructions. Protein coated beads were incubated with BSA (100 µg/ml) in order to block the non-specific binding.

Fly head lysates were prepared by homogenizing 50 mg of fly heads in 1 ml of lysis buffer (20 mM Tris Cl; pH 7.5, 100 mM NaCl, 0.5% NP40, 0.5 mM EDTA, 0.5 mM PMSF and EDTA-free complete protease inhibitor tablets (Roche). The homogenate was centrifuged at 13,000 rpm at 4°C for 30 min. The supernatant was removed and precleared with glutathione-agarose beads for 1 h. Equal volumes of supernatant were then incubated with either 5 µg of GST coated beads or 5 µg various GST-Synd domain recombinant protein-coated beads for 1 h with end-over-end rotation at 4°C. Beads were then washed 8-10 times with the lysis buffer and boiled in Laemilli buffer (2% SDS; 10% glycerol; 62.5 mM Tris-Cl, pH 6.8; 100 mM DTT, 0.1% bromophenol blue). The eluted proteins were resolved on 12% SDS-PAGE followed by Western transfer. Wsp pulled down by various domains of Syndapin was ascertained by probing the blot with anti-Wsp primary antibody and HRP coupled secondary antibody using ECL detection system (Amersham, Arlington Heights, IL).

In order to pull down Syndapin, the proline rich domain (PRD) of Shibire was amplified using PCR primers: ShiPRDfor (5′-CGCGGATCCTCGCCATTGCCACCG-3′) and ShiPRDrev (5′-CCCAAGCTTTTAACGACGCGATGGTAGC-3′) and ligated in pGEX-KG vector in BamHI and HindIII restriction sites to express as a GST-fusion protein. The pull down was performed as above for Wsp. Eluted proteins were resolved as described above. Syndapin pulled down by GST-ShiPRD was ascertained by probing the blot with anti-Synd primary antibody; HRP coupled secondary antibody and detection using ECL.

###### Western blotting

Fly heads were homogenized in 1X SDS sample buffer (50 mM Tris-Cl; pH 6.8, 2% SDS, 2% β-ME, 0.1% bromophenol blue, 10% Glycerol), boiled for five min and 2 fly head equivalent of protein was fractionated on a 13.5% SDS PAGE. The protein was transferred onto PVDF membrane and blocked for 1 h in 5% fat-free milk. The rat anti-Syndapin antibodies were used at 1:10,000 dilutions. The anti-rat HRP antibody (Amersham Pharmacia Biotech) was used at 1:5000 dilutions. Signals were detected using ECL system.
